# The Oncopig as an Emerging Model to Investigate Copper Regulation in Cancer

**DOI:** 10.3390/ijms232214012

**Published:** 2022-11-13

**Authors:** Alyssa L. Carlson, Jaime Carrazco-Carrillo, Aaron Loder, Lobna Elkhadragy, Kyle M. Schachtschneider, Teresita Padilla-Benavides

**Affiliations:** 1 Department of Molecular Biology and Biochemistry, Wesleyan University, Middletown, CT 06459, USA; 2 Department of Radiology, University of Illinois at Chicago, Chicago, IL 60607, USA; 3 Department of Biochemistry and Molecular Genetics, University of Illinois at Chicago, Chicago, IL 60607, USA; 4 National Center for Supercomputing Applications, University of Illinois at Urbana-Champaign, Urbana, IL 61820, USA

**Keywords:** copper, Oncopig, homeostasis, metal transport, cuproproteins

## Abstract

Emerging evidence points to several fundamental contributions that copper (Cu) has to promote the development of human pathologies such as cancer. These recent and increasing identification of the roles of Cu in cancer biology highlights a promising field in the development of novel strategies against cancer. Cu and its network of regulatory proteins are involved in many different contextual aspects of cancer from driving cell signaling, modulating cell cycle progression, establishing the epithelial-mesenchymal transition, and promoting tumor growth and metastasis. Human cancer research in general requires refined models to bridge the gap between basic science research and meaningful clinical trials. Classic studies in cultured cancer cell lines and animal models such as mice and rats often present caveats when extended to humans due to inherent genetic and physiological differences. However, larger animal models such as pigs are emerging as more appropriate tools for translational research as they present more similarities with humans in terms of genetics, anatomical structures, organ sizes, and pathological manifestations of diseases like cancer. These similarities make porcine models well-suited for addressing long standing questions in cancer biology as well as in the arena of novel drug and therapeutic development against human cancers. With the emergent roles of Cu in human health and pathology, the pig presents an emerging and valuable model to further investigate the contributions of this metal to human cancers. The Oncopig Cancer Model is a transgenic swine model that recapitulates human cancer through development of site and cell specific tumors. In this review, we briefly outline the relationship between Cu and cancer, and how the novel Oncopig Cancer Model may be used to provide a better understanding of the mechanisms and causal relationships between Cu and molecular targets involved in cancer.

## 1. Introduction

Copper (Cu) is a trace element essential to the development of mammalian cells and tissues due to its chemical properties and redox potential. Cu is necessary for many vital and diverse biological processes, such as cellular respiration, maintenance of redox homeostasis, maturation of collagens and elastins, synthesis of melanin and catecholamine, processing of neuro-hypophyseal hormones, and transcriptional regulation [1,2,3,4,5]. Despite its importance, Cu can also be toxic in greater quantities. This potential toxicity requires that Cu levels be highly regulated on both a cellular and systemic level. Mammalian cells have developed a complex network of transporters, chaperones, and transcriptional regulators to preserve Cu homeostasis [6,7,8,9,10,11]. Physiologically, the ion exists as Cu^+^ and Cu^2+^. While the monovalent form is the principal state found in the cytosol, some cuproenzymes take advantage of the redox potential of the metal to function. Classic examples of redox active cuproenzymes are cytochrome c oxidase (COX), the final electron acceptor metalloenzyme of the respiratory chain [12], and superoxide dismutase (SOD1 and SOD3), which scavenge toxic radicals to maintain cellular redox homeostasis both within the cell and in the extracellular matrix (ECM) [13].

Cu is part of numerous biological processes, some of which may lead to enhanced cell proliferation, energy production, and fitness to survive [14,15]. These events can contribute to the onset, development, and progression of pathological conditions such as cancer. Research demonstrates that Cu plays an important and yet underappreciated role in carcinogenic processes. A recent publication by Ge and collaborators coined the novel term “cuproplasia” to represent the exacerbated growth and proliferation of cells or tissues in the body in a Cu-dependent manner [16]. Cuproplasia defines both the direct and indirect effects that Cu triggers in carcinogenic signaling pathways, which may include enzymatic and non-enzymatic Cu-dependent processes [16]. Previous studies corroborate this result. Proliferating cancer cells were found to have a higher requirement for Cu than non-cancerous cells [17]. Restricting Cu availability to cancer cells has been shown to reduce the progression of the disease [18]. An early study by Ishida and collaborators showed that chronic exposure to elevated levels of Cu in drinking water promoted the proliferation of cancer cells and development of pancreatic tumors in mice [19]. Furthermore, the authors showed that limiting bioavailable Cu on the systemic level with chelating agents impaired these carcinogenic processes due to a decrease in the activity of COX, and by consequence, less ATP for the cells to utilize [19]. Due to the importance of Cu in the regulation of cellular processes relevant to cancer, there is an increased scientific interest in understanding the molecular link between Cu homeostasis and cancer development, as well as the potential manipulation of this metal as part of anti-cancer therapies [20].

## 2. The Regulatory Machinery for Cu Homeostasis

**Figure 1:**
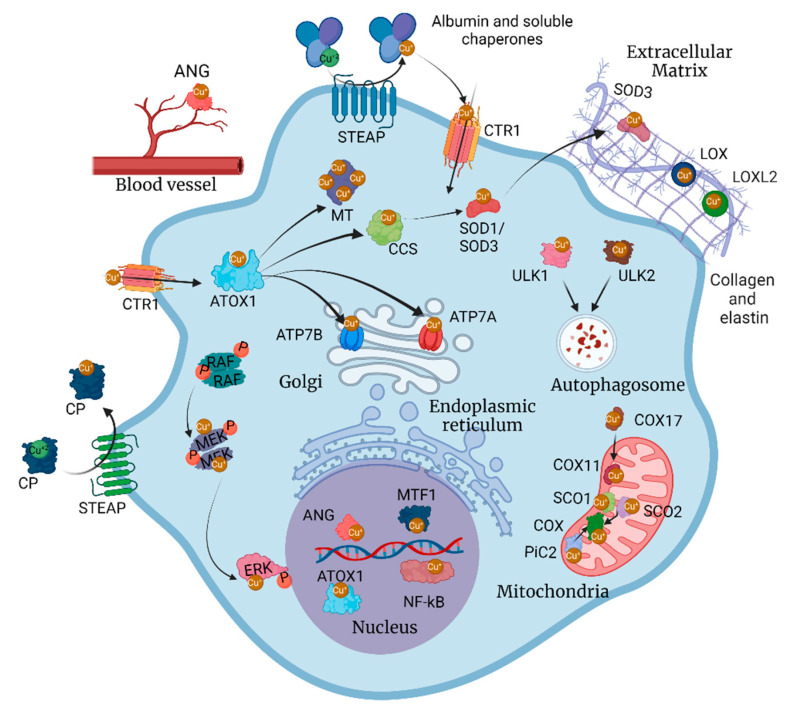
The copper (Cu) network in cancer. Carcinogenic cellular pathways like cell proliferation, survival, migration, invasion, metastasis, and drug resistance are enhanced by Cu bioavailability. Cu-responsive molecules are involved in cell cycle, angiogenesis, remodeling of the extracellular matrix, and autophagy. Figure created with BioRender.com (accessed on 10 October 2022).

Cellular Cu homeostasis is regulated by a complex network of transmembrane transporters, storage proteins, metallochaperones, and transcription factors (Figure 1). In plasma, Cu is transported primarily by ceruloplasmin (CP) to reach tissues and organs where the metal ion is required. CP, a Cu-dependent iron (Fe) oxidase, binds Cu^2+^ and systemically delivers the ion to individual cells [21]. At the cell surface, CP interacts with six-transmembrane epithelial antigen of the prostate (STEAP) proteins, a family of metalloreductases that reduces divalent Cu^2+^ to its monovalent form [22] and allows its internalization by copper transporter 1 (CTR1) [23]. Within the cell, various metallochaperones control the distribution and delivery of cytosolic and mitochondrial Cu to their subcellular compartments and final acceptors. Antioxidant protein 1 (ATOX1) mediates Cu secretion by interacting with the Cu^+^-ATPases ATP7A and ATP7B in the *trans* Golgi network, but also potentially with additional, yet unknown, cellular components [24,25,26,27,28,29]. ATOX1 can also interact with and deliver Cu to the Cu chaperone for superoxide dismutase (CCS), which ultimately delivers the ion to superoxide dismutase (SOD1 and SOD3) to control oxidative stress induced by hydroxyl radicals [13,30,31,32,33]. COX17 mobilizes the metal into the mitochondria, where other chaperones and reductases SCO1, SCO2, and COX11 ensures delivery to the COX complex [8,34,35,36,37,38,39,40]. The metallothioneins (MT1-4) are tasked with the storage of the metal ion within the cell, regulating its availability [41,42,43]. The Cu^+^-ATPases ATP7A and ATP7B export the ion to the ECM and blood, bound to secreted cuproproteins containing methionine, cysteine, histidine, aspartic acid, and glutamic acid residues and soluble carriers such as CP and albumin, [44,45]. Finally, transcription factors like MTF1 and nuclear ATOX1 control the expression of these Cu^+^ transporters and chaperones to ensure that cells maintain their fundamental Cu needs [1,5,46,47,48,49].

## 3. Copper in Cancer

Excessive proliferation, migration, invasion, and evasion of apoptotic pathways are central hallmarks of cancer and are mediated by dysregulated signaling and transcriptional modulation that changes the fundamental biology of cells [50,51]. These pathways have a strong connection with Cu biology and homeostasis, making this ion and its regulatory network potential targets for treatments that mitigate cancer development and progression [15,16,52,53,54,55,56,57,58]. Cancer cells have increased requirements for Cu, as the ion plays a role in the establishment of the carcinogenic phenotype. For instance, the increased demand for Cu by cancer cells is partially due to the increased COX activity that helps sustain the increase in cell proliferation [58]. Other hallmarks of cancer, such as ECM remodeling, autophagy, migration, and angiogenesis, also require activation of cuproenzymes and Cu-dependent pathways to facilitate Cu import, cuproprotein synthesis, and cuproenzyme catalytic activity [55,59]. This is corroborated by the detection of increased Cu levels in the tissues and serum of cancer patients (Reviewed in [16,17]). In addition, an increased occurrence of cancer in Wilson Disease patients has been reported. Wilson Disease patients have an impaired ability to excrete excess systemic Cu, which establishes an environment that is favorable for the onset and progression of cancer [16].

Other Cu-driven effects in signaling pathways involved in cancer progression are starting to be uncovered. For instance, Cu modulates cyclic AMP (cAMP) metabolism, thereby regulating G-protein-coupled receptor (GPCR) signaling, in a lineage-specific manner [60,61]. A study in Chinese hamster ovary fibroblasts evaluating activity of Melanocortin-4 receptors (MC4R) showed that Cu is a negative allosteric modulator of ligand binding to this receptor [60]. Additionally, in white adipocyte cells, Cu potentiates the activity of GPCRs and inhibits cAMP degradation by binding the cAMP degrading enzyme phosphodiesterase 3B (PDE3B), resulting in an increase in cAMP levels [61]. The AKT signaling pathway, which mediates cell growth and survival, has also been shown to be activated by Cu transport via CTR1 modifying the upstream effector phosphoinositide-dependent kinase-1 (PDK1) [62]. Cu also serves as an activator of the Mitogen-activated protein kinase kinase 1 and 2 (MEK1 and MEK2, respectively), key intermediaries in the RAF (rapidly accelerated fibrosarcoma) protein kinase signaling pathway [15,57]. In vitro studies have shown that Cu supplementation activates additional mitogen-activated protein kinase (MAPK) pathway members—e.g., Tropomyosin receptor kinase B (TRKB), epidermal growth factor receptor (EGFR), and mesenchymal–epithelial transition factor (MET)—in cortical neurons and in the human epithelial and lung cell lines DU145 and A549, respectively, even in the absence of their ligands [63,64]. Cu activates the Unc-51 like autophagy activating kinases 1 and 2 (ULK1 and ULK2, respectively), which favor the breakdown of various subcellular components as a means of providing energy to cells. The activation of ULK1 and ULK2 by Cu enhances energy production in cancer cells, which sustains the energetic cost of continuous cell growth and division [55]. These non-redundant kinases may also provide energy to cells under nutrient-deficient conditions, as is found in the center of a tumor, further aiding the survival of cancer cells, and allowing for their continual proliferation [65]. All these pathways are major drivers of cell cycle progression and proliferation, which highlights the impact of Cu on critical processes during cancer development.

Although elevated intracellular Cu levels do not appear to be a direct cause of cancer, excessive Cu plays a key role in driving epithelial-mesenchymal transition (EMT), ECM remodeling and carcinogenesis. EMT is a reversible trans-differentiation program where epithelial cells acquire a mesenchymal phenotype with more plasticity and dynamism [66]. EMT is a normal process essential for embryonic development, wound healing and tissue regeneration; however, it is also observed in the development of cancer [66]. EMT is marked by the loss of epithelial markers (E-cadherin, Zonula occludens-1 and claudins) and the expression of mesenchymal proteins (N-cadherin, vimentin and matrix metalloproteases (MMPs)). This is caused by the dysregulation of the expression and activity of transcription factors such as Snail, Slug, Zeb and Twist mediated by a number of upstream signaling cascades [66], some of which are activated by Cu, as discussed above. Therefore, the resulting phenotype is characterized by the alteration of the expression of genes required for maintenance of adhesion, polarity and cytoskeleton reorganization [67].

In addition, Cu contributes to the modification of ECM, as this ion serves as an activator for lysyl oxidases (LOX) and lysyl-oxidase like proteins (LOXL). These proteins are prevalent in invasive cancer cells as they are required for remodeling the surrounding ECM [59,68,69,70,71,72,73,74,75,76,77,78,79,80,81,82]. The formation of the tumor stroma requires remodeling and stiffening of the ECM [83,84]. ECM stiffness promotes cell proliferation, survival, and migration; this rigidity disrupts formation of tissue morphogenesis due to increased cell tension [85,86]. There are still gaps on the knowledge on the mechanisms underlying ECM stiffening and changes in tension promote tumor progression. Among the most relevant ECM proteins is collagen as it provides strength and tension to the tissues [87]. In cancer, the metabolism of collagen is altered in cancer, and is characterized by increased collagen synthesis, accumulation, organization. Increased activity of zinc dependent MMPs and a consequent collagen remodeling may also favor migration of metastatic cells accelerating tumor progression [88]. Moreover, the secreted Cu-activated LOX and LOXL oxidases catalyze lysine oxidation in elastins and collagens which results in crosslinking that provides structure, stability, and stiffness to the ECM scaffolding [71,89]. Increased LOX expression and enhanced stiffness in the tumor microenvironment are both associated with cancer progression and metastasis in solid tumors such as breast, colorectal, and prostate cancers [69,71]. To form secondary tumor sites, ECM remodeling events are necessary to induce EMT. LOX and LOXL proteins are proposed to promote EMT in cancer cells, and given Cu is a co-factor for their activity, this suggests that elevated Cu levels could serve as a driver of carcinogenesis [59]. Cu may also be an allosteric modulator of the activity of the ubiquitin-conjugating enzyme E2 D (UBE2D). A conserved CXXXC motif in this protein acts as a Cu^+^-binding motif with sub-femtomolar-affinity. Variations in cytoplasmic concentrations of Cu^+^ may promote the degradation of UBE2D targets, such as p53 [90]. This is supported by data showing that a Cu-rich environment supports the degradation of p53, removing this key checkpoint player in regulating cell division and preventing the accumulation of mutations, which can greatly increase the risk of cancer [90].

Angiogenesis is another crucial aspect in tumor development, and Cu has been associated with blood vessel formation and endothelial cell migration [19,91,92,93,94]. These processes are fundamental for metastasis to occur, as the formation of new blood vessels is often followed by cancer cell migration to establish secondary tumor sites. Cu stimulates transcription factors involved in vessel formation and maturation, such as the hypoxia-inducible factor-1 (HIF-1). HIF-1 regulates the expression of the vascular endothelial growth factor (VEGF), which is a main driver of angiogenesis. Cu has been proposed to be transported into nucleus by a CCS, and potentially delivered to HIF-1. Metallated HIF-1 interacts with the hypoxia-responsive element of the target genes and enables transcription, including that of VEGF. Furthermore, Cu stabilizes and promotes accumulation of the HIF-1α subunit, favoring HIF-1 activation [95,96]. In addition, the angiogenic growth factor, angiogenin (ANG) also binds and is activated by Cu, and positively regulates Fibroblast Growth Factor (FGF) and interleukin 1α (IL-1α) secretion and further promotes the formation of new blood vessels. Another mechanism by which Cu enhances angiogenesis is through the activation of the NF-κB signaling pathway, which induces the expression of pro-angiogenic factors such as VEGF, bFGF, IL-1α, IL-6, and IL-8 [94,95,97,98,99,100,101,102] facilitating the formation of secondary tumors, and is a notable effect of this metal ion.

The ability of Cu to facilitate cellular respiration through COX function and promotion of autophagy through ULK1 and ULK2 activation enhances cancer cell survival. Cu plays an indirect role in the promotion of cell proliferation by inhibiting the degradation of cAMP, effectively activating GPCR pathways. However, Cu also contributes to the dysregulation of cell cycle progression by activating the MEK1 and MEK2 proliferative signaling pathways. The role of Cu as a regulator of UBE2D1-4 also promotes the degradation of p53, which can result in the accumulation of carcinogenic mutations through removing a key cell cycle checkpoint. Finally, Cu serves as an activator of ECM remodelers, allowing the establishment of the EMT, angiogenesis, and the consequent spread of cancer throughout the body at later stages of disease. Thus, various elements of the Cu network serve as emerging targets for cancer drugs and therapies that reduce the intracellular concentration and bioavailability of the ion in cancer cells. To this end, Cu chelators have been utilized to reduce Cu bioavailability, resulting in a decrease in cell proliferation and, by consequence, the progression of cancer. Chelating agents (e.g., tetrathiomolybdate and D-penicillamine), as well as Cu ionophores (e.g., disulfiram and Elesclomol), are used in combination with other anti-cancer drugs to either reduce systemic Cu levels or induce cuproptosis (copper-induced cell death [103]) as novel therapies against various types of carcinomas. Their effects and mechanisms of action have been reviewed elsewhere [10,16].

## 4. Investigating Cu Regulators in a Porcine “Oncopig” Cancer Model

Cumulative evidence highlights novel roles for several cuproproteins in cancer progression and metastasis. However, more translational studies are required to elucidate the fine mechanisms of action of these molecules in the context of human cancers. Animal models are important tools for investigating molecular mechanisms and testing therapeutic strategies against cancer. The development of an inducible porcine model for human cancer, known as the “Oncopig Cancer Model” (OCM) offers a novel and favorable platform for studying human cancer in a large animal model [104,105,106,107,108]. Due to the pig’s high similarity to humans regarding size, genetic homology, metabolism, physiology, and pathology, the Oncopig likely offers more accurate representation of human cancers than other animal models [105,109,110,111,112,113,114,115]. The use of this porcine model to investigate cancer may provide more predictive health outcomes and clinical advantages through optimized screening of potential cancer treatments, thus providing a higher predictability of how therapeutics will perform in human oncological clinical trials [116,117,118].

**Figure 2:**
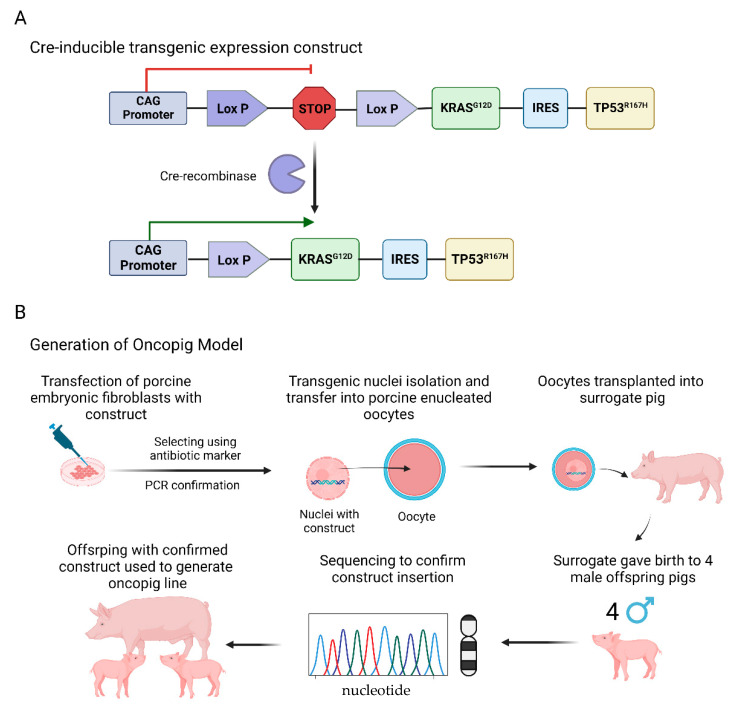
Development of the Oncopig model. (**A**) Development of the Cre-inducible transgene construct. (**B**) Steps used for creation of the Oncopig cancer model. Figure created with BioRender.com.

The Oncopig harbors a Cre recombinase inducible expression construct for porcine KRASG12D, which is a constitutively active KRAS, as well as TP53R167H, a dominant negative TP53 mutant [107,108]. Exposure to adenoviral vectors encoding Cre recombinase (AdCre) drives the inducible expression of the two oncogenic transgenes, which allows for reproducible in vitro and in vivo transformation of Oncopig cells. The Oncopig cancer model has been shown to recapitulate several human cancers including soft tissue sarcomas (STS) of mesenchymal origin developing from intramuscular regions, hepatocellular carcinoma (HCC), and others. These tumors developed in Oncopigs emulate human cancers in terms of transcriptional hallmarks, cytological features, and histology (Figure 2; [104,106,107,108]). Additional Oncopig cancer cell lines and refined in vivo models have been generated since the original development of this transgenic animal. However, despite the advances in the research derived from this model, its usefulness as a tool to investigate a variety of pathogenic processes that occur in cancer remain unexplored.

Given that tumors developed in Oncopigs recapitulate human cancers, the Oncopig is an excellent model to investigate the contributions of Cu and its related proteins in the development of cancer. We performed a review and bioinformatics analysis of published transcriptomic datasets obtained from Oncopig STS tumors that were compared to bulk skeletal muscle tissue (ArrayExpress accession number E-MTAB-3382), as well as data from Oncopig HCC cells which were compared with normal porcine primary hepatocytes (European Nucleotide Archive accession number PRJEB8646). We focused our transcriptional analysis to known Cu-binding proteins [107,108] and identified cuproproteins demonstrating differential expression in Oncopig-derived STS and HCC as compared to their respective controls. The Oncopig is a novel and unexplored model that has a remarkable potential to contribute to our knowledge on the emerging mechanisms by which Cu contributes to cancer progression.

## 5. Differential Expression of Cu-Related Genes in Oncopig Soft Tissue Sarcoma

**Figure 3:**
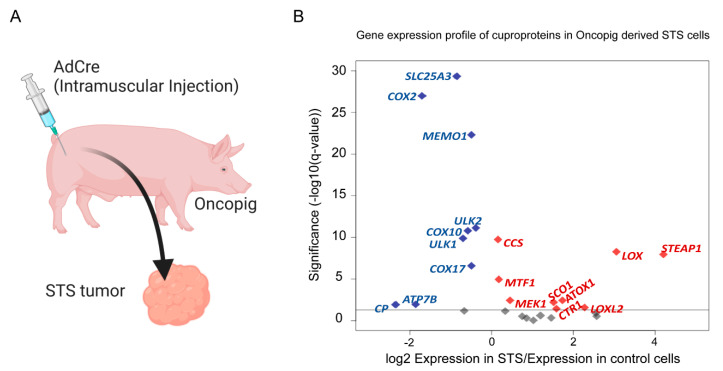
Differential expression of Cu-related genes in Oncopig-derived STS Cells. (**A**) Process to induce Oncopig STS tumors. (**B**) Volcano plot of genes encoding cuproproteins in Oncopig STS cells, plotting magnitude expression difference by significance. The horizontal line represents q-value = 0.05. Red indicates significant upregulation, blue denotes significant downregulation, and grey represents non-significant changes in expression. STS Accession codes: The data sets supporting the results of this review article are available in ArrayExpress accession number: E-MTAB-3382. Figure created in BioRender.com and R software (version 4.0.3) [125].

Oncopig STS tumors were generated by intramuscular injection of AdCre into the hind limb skeletal muscle which resulted in the formation of tumors that resemble human STS characteristics [108]. The published RNA-seq datasets for STS tissues from the OCM showed that nineteen Cu-binding genes were differentially expressed. Of these, seven genes were significantly upregulated and eleven were downregulated. Out of these differentially regulated genes, STEAP1 had the highest level of upregulation (Figure 3). This Fe/Cu metalloreductase is implicated in the metabolism of reactive oxygen species (ROS) and reduces extracellular Cu^2+^ to Cu^+^ to enable Cu import into the cell via CTR1 [119]. Interestingly, STEAP1 expression is known to be upregulated in Ewing sarcoma, prostate cancer, and breast cancer [120,121,122]. In human Ewing sarcoma cell lines, STEAP1 knockdown decreases proliferation, invasion, growth, and metastatic potential, suggesting a crucial role for STEAP1 in tumorigenic properties in patient-derived Ewing sarcoma cell lines [122]. MEK1, a Cu-binding kinase that is part of the MAPK and ERK signaling pathways, is also upregulated in STS tumors [54]. In BRAF^V600E^-positive melanomas, the mutated BRAF kinase drives the activation of MEK1/2 and subsequently ERK1/2 which stimulates the MAPK pathway [54]. MEK1/2 and BRAF^V600E^ inhibitors have risen as widely used therapeutic options to reduce tumorigenesis in these melanomas [54]. ECM remodeling genes LOX and LOXL2 were also highly upregulated in the Oncopig STS model (Figure 3). As discussed above, LOX and LOXL2 encode homologous secreted Cu-dependent amine oxidase proteins that are metalated by ATP7A and are required for ECM remodeling [59]. MTF1 is another upregulated gene as well, which encodes a transcription factor that regulates the expression of numerous genes including Cu^+^-transporters and chaperones required to maintain metal and redox homeostasis and is also required for skeletal muscle differentiation [1,5,123]. Other significantly upregulated genes encoding cuproproteins in the Oncopig STS model include CTR1 and ATOX1, further implicating enhanced Cu internalization and cytosolic transport in this type of cancer. CCS, which is metalated by ATOX1 and is a Cu chaperone for SOD1 was upregulated as well [30,32]. Furthermore, an in vitro study showed that CCS may promote proliferation and migration in breast cancer cells [124]. SCO1 was also among the upregulated genes, and its role as a mitochondrial Cu chaperone and COX assembly protein suggests Cu plays a potential role in mitochondrial function in cancer. Eight Cu-binding genes were significantly downregulated in the STS model, and among these were the systemic Cu carrier CP and the autophagy kinases ULK1, and ULK2. Mitochondrial genes that were downregulated include COX2, COX10, COX17, and SLC25A3 (PiC2). Additionally, MEMO1 a Cu-dependent cell motility mediator was also downregulated (Figure 3). The numerous changes in the expression of Cu-binding proteins in the Oncopig STS model strongly argues in favor of the need for more experimental studies characterizing the role of Cu in human cancers.

## 6. Differential Expression of Cu-Related Genes in Oncopig Hepatocellular Carcinoma

**Figure 4:**
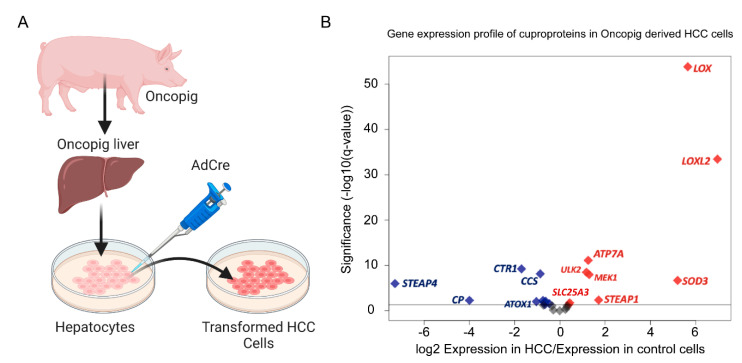
Differential expression of Cu-related genes in Oncopig-derived HCC cells. (**A**) Process to obtain Oncopig HCC cell lines. (**B**) Volcano plot of genes encoding for cuproproteins in Oncopig HCC cells plotting magnitude expression difference by significance. The horizontal line represents q-value = 0.05. Red indicates significant upregulation, blue denotes significant downregulation, and grey represents non-significant changes in expression. HCC Accession codes: The datasets are available in the European Nucleotide Archive accession number: PRJEB8646. Figure created in BioRender.com and R software (version 4.0.3) [125].

Porcine HCC cell lines were developed by isolating Oncopig primary hepatocytes and inducing oncogenic transgene expression by AdCre exposure resulting in HCC cells that recapitulated human HCC phenotypes [106,107]. Autologous injection of these transformed cells into Oncopig livers resulted in intrahepatic liver tumor development [106,126]. By transcriptional analysis of Oncopig HCC cells as compared to normal Oncopig primary hepatocytes, we identified nineteen genes encoding for cuproenzymes that were differentially expressed. Among these, eight genes were significantly upregulated and eleven were downregulated (Figure 4). Similar to STS, the Cu-dependent collagen and elastin remodeling oxidases LOX and LOXL2 were among the genes with the largest induction in expression in the Oncopig-derived transformed HCC cells compared to untransformed Oncopig hepatocyte controls. This corroborates another study that reports increased expression of LOXL2 in human HCC tumors [77]. Functionally, LOX and LOXL2-dependent cross-linking of collagen and elastin has many roles, including increasing tissue stiffness and strength, promotion of cancer cell organization, activation of growth factors, and facilitation of matrix metalloprotease activity. These processes are well-known to participate in the establishment of pre-metastatic niches in areas outside of the primary tumor site, as observed in numerous human cancers such as HCC, colorectal cancer, breast cancer, and gastric cancer [68,75,77,79,81,82]. Relatedly, ATP7A expression was induced, and given it functions in the regulation of Cu transport across membranes, this suggests an exacerbated and potentially more aggressive mechanism to metallate and promote Cu-mediated LOX activation. SOD3 is another upregulated gene in Oncopig-derived HCC cells. SOD3 binds Cu and Zn to catalyze extracellular detoxification of ROS to protect cells from oxidative stress [127]. Other significantly upregulated genes encoding Cu-binding proteins include STEAP1, ULK2, and MEK1. Among the mitochondrial genes upregulated was the Cu^+^ transporter SLC25A3 (PiC2).

Conversely, downregulated cuproprotein genes in the Oncopig HCC cell line included the Cu/Fe metalloreductase STEAP4 (Figure 4), which is involved in adipocyte, hepatocyte, and pancreatic cell development and metabolism and has been implicated to play a role in inflammatory stress response [128]. The decrease of STEAP4 in Oncopig HCC cells is consistent with studies in human HCC tumors showing that the STEAP4 promoter is silenced via methylation [129]. CP, a Cu carrier protein that is synthesized in the liver, was down-regulated in the Oncopig HCC (Figure 4A,B). This contradicts serological studies in a murine transgenic model for HCC expressing viral oncogene SV40-Tag that reported an increased level of CP in serum [130], as well as observations in the serum of patients with solid tumors of the lung, breast, head/neck, and gastrointestinal tract [131]. However, low CP levels in the serum have been reported in human patients with viral hepatitis-associated liver disease, drug and alcohol-induced liver disease, and Wilson disease [132,133,134], suggesting a specific regulation of this gene in the liver, which correlates with the maturation state of the HCC cells used in the original study. Additional significantly downregulated Cu-binding genes in Oncopig HCC include CTR1, CCS, ATOX1, COX2, COX17, COX10, and MTF1.

The variations observed in the gene expression profiles of these two different Oncopig-derived cancer models, HCC and STS, suggest differential contributions from components of the Cu regulatory network in the establishment and development of different carcinogenic phenotypes. Overall, our review of published Oncopig RNA-seq datasets and bioinformatic analyses showed consistencies in the variability of expression of cuproenzymes in human cancer, highlighting the importance of this novel porcine model in the study of cancer and metals in the pathophysiology of disease. However, some discrepancies were found between STS and HCC cuproenzymes, including ULK2, CTR1, CCS, ATOX1, MTF1, and SLC25A3 (PiC2). The differential expression of these proteins and downstream targets may be also a consequence of the different nature of the carcinomas and sarcomas as well as differences between in vivo and in vitro cancer models. The Oncopig HCC results represent analyses of in vitro primary HCC cell line expression, compared to in vitro normal hepatocytes, both derived from the Oncopig liver. The STS data corresponds to in vivo sarcoma tumors compared to in vivo normal skeletal muscle, both harvested directly from the animal. Therefore, in addition to representing two different cancer types, the HCC cell line data represents expression of “pure tumor cells”, whereas the STS tumor expression results may represent a combination of tumor and non-tumor (stroma) cell populations. Furthermore, in terms of the genes that showed opposite differential expression between the two cancer types, there is also controversial or limited evidence available. For example, there is no clear evidence of the contributions of ULK2 to STS progression, thus the role for this protein in STS patients remains unknown, as limited information in general is available for this cancer type. On the other hand, the enhanced expression of ULK2 observed in HCC Oncopig samples are comparable to published analyses of HCC patients datasets obtained from The Cancer Genome Atlas Program (TCGA; GSE54236 dataset) [135]. This study showed that patients from low-risk HCC groups have a significantly higher expression of ULK2, suggesting that autophagy may play a protective role in the low-risk group compared to high-risk individuals [136]. The role for this protein in STS patients remains unknown, as limited information is available for this cancer type. Thus, the analyses presented here further supports the Oncopig model as an excellent tool to answer these questions when patient information is not accessible. Hence, systematic studies of these and other tissues will shed light on the contributions of the various members of the Cu network in the onset and development of cancer. The biological similarities encountered between porcine models and human biology confers the Oncopig a number of potential applications for research oriented towards understanding the biological function of Cu and establishes it as a powerful tool for the development of potential therapies, including Cu chelating drugs, in the treatment of human cancer.

## 7. Conclusions

Cu is a trace metal fundamental for cellular respiration, cell signaling, cell cycle progression, remodeling of the ECM, and promotion of angiogenesis. Increasing evidence points to the sophisticated strategies that cancer cells have developed to acquire and use this metal ion at levels that favor cell division, metastasis, and other carcinogenic features. Cu metabolism in cancer cells is an emerging field of research that could shed light onto novel strategies to combat this complex disease. Research shows that Cu signaling can be regulated by using specific chelators and ionophores, as well as by genetic manipulation of the proteins controlling Cu homeostasis. The Oncopig presents a well-suited model for the study of human cancer, including the contributions of Cu in malignant transformation. Our transcriptomic analysis suggests many Cu-related molecular changes in an Oncopig model of STS and HCC. Research should be directed to utilize novel and powerful animal models like the Oncopig, as this confers higher accuracy to human cancer and its connection to copper biology. Considering that the only work done in this area is the gene expression analysis presented in this review, there is great potential to elucidate novel paradigms related to cuproplasia using the Oncopig model. In conclusion, the biological similarities with human pathology place the Oncopig model as a powerful tool for advancing our understanding the mechanisms and causal relationships between Cu and molecular targets involved in cancer.

## Data Availability

Previously published datasets utilized in the Review are available at ArrayExpress (accession number E-MTAB-3382) and the European Nucleotide Archive (accession number PRJEB8646).
